# Age, sex, endurance capacity, and chronic heart failure affect central and peripheral factors of oxygen uptake measured by non-invasive and continuous technologies: support of pioneer work using invasive or non-continuous measures

**DOI:** 10.3389/fspor.2023.1218948

**Published:** 2023-09-05

**Authors:** Joana Brochhagen, Michael T. Coll Barroso, Christian Baumgart, Daniel T. Wasmus, Jürgen Freiwald, Matthias W. Hoppe

**Affiliations:** ^1^ Movement and Training Science, Faculty of Sport Science, Leipzig University, Leipzig, Germany; ^2^ HELIOS Klinikum Wuppertal, University of Witten/Herdecke, Wuppertal, Germany; ^3^ Department of Movement and Training Science, Faculty of Humanities and Social Sciences, Institute of Sport Science, University of Wuppertal, Wuppertal, Germany

**Keywords:** catheter, exercise physiology, exercise testing, haemodynamics, new technologies

## Abstract

**Introduction:**

It is known that maximum oxygen uptake depends on age, sex, endurance capacity, and chronic heart failure. However, due to the required invasive or often applied non-continuous approaches, less is known on underlying central and peripheral factors. Thus, this study aimed to investigate the effects of age, sex, endurance capacity, and chronic heart failure on non-invasively and continuously measured central and peripheral factors of oxygen uptake.

**Methods:**

15 male children (11 ± 1 years), 15 male (24 ± 3 years) and 14 female recreationally active adults (23 ± 2 years), 12 male highly trained endurance athletes (24 ± 3 years), and 10 male elders (59 ± 6 years) and 10 chronic heart failure patients (62 ± 7 years) were tested during a cardiopulmonary exercise test on a cycling ergometer until exhaustion for: blood pressure, heart rate, stroke volume, cardiac output, cardiac power output, vastus lateralis muscle oxygen saturation, and (calculated) arterio-venous oxygen difference. For the non-invasive and continuous measurement of stroke volume and muscle oxygen saturation, bioreactance analysis and near-infrared spectroscopy were used, respectively. A two-factor repeated measure ANOVA and partial eta-squared effect sizes ($\eta_{p}^{2}$) were applied for statistical analyses at rest, 80, and 100% of oxygen uptake.

**Results:**

For the age effect, there were statistically significant group differences for all factors (*p* ≤ .033; $\eta_{p}^{2}\geq .169$). Concerning sex, there were group differences for all factors (*p* ≤ .010; $\eta_{p}^{2}\geq .223$), except diastolic blood pressure and heart rate (*p* ≥ .698; $\eta_{p}^{2}\leq .006$). For the effect of endurance capacity, there were no group differences for any of the factors (*p* ≥ .065; $\eta_{p}^{2}\leq .129$). Regarding chronic heart failure, there were group differences for the heart rate and arterio-venous oxygen difference (*p* ≤ .037; $\eta_{p}^{2}\geq .220$).

**Discussion:**

Age, sex, endurance capacity, and chronic heart failure affect central and peripheral factors of oxygen uptake measured by non-invasive and continuous technologies. Since most of our findings support pioneer work using invasive or non-continuous measures, the validity of our applied technologies is indirectly confirmed. Our outcomes allow direct comparison between different groups serving as reference data and framework for subsequent studies in sport science and medicine aiming to optimise diagnostics and interventions in athletes and patients.

## 1. Introduction

Exercise capacity reflects the maximum amount of physical exertion a person can achieve (1). Its assessment is established in different settings, ranging from the evaluation of physical performance in athletes (2) to cardiorespiratory diseases in patients (3). Thereby, the assessment of maximum oxygen uptake during cardiopulmonary exercise testing is considered as the gold-standard approach (4). The oxygen uptake is defined by the Fick's equation as the product of cardiac output and arterio-venous oxygen difference (5). Thus, oxygen uptake depends on several central and peripheral factors, including the stroke volume and heart rate as well as mixed arterio-venous oxygen content, respectively (5). It is well known that maximum oxygen uptake depends on age, sex, endurance capacity, and diseases clearly limiting exercise capacity such as chronic heart failure (6–8). However, more research accounting for the underlying physiological factors on a central and peripheral level is needed.

Maximum oxygen uptake must be understood as a surrogate parameter, reflecting the interaction of the respiratory, cardiovascular, and muscular system (6). As maximum oxygen uptake is influenced by several factors (6), its estimation alone does not allow any clear conclusions to be drawn about the efficiency of the cardiopulmonary system and muscle tissue (9) or underlying causes of exercise intolerance (10). Therefore, it is essential to take central and peripheral factors of oxygen uptake into account for optimising diagnostics and interventions in both athletes and patients (9, 11). Due to the invasive or often non-continuous character of established technologies such as pulmonary artery catheter thermodilution or transoesophageal echocardiography and rebreathing technique or transthoracic echocardiography (12), respectively, new non-invasive and continuous approaches are promising to apply in research and practice (11).

In this context, thoracic bioreactance analysis and near-infrared spectroscopy are such technologies to estimate central and peripheral factors of oxygen uptake in a non-invasive and continuous manner (13, 14). In fact, cardiac output reflects the haemodynamic response to physical exercise in healthy participants (15) and patients (16), and cannot be predicted from maximum oxygen uptake alone (17, 18). However, the stroke volume can be measured using thoracic bioreactance analysis (14). Then, cardiac output using stroke volume and heart rate as well as cardiac power output additionally using (mean arterial) blood pressure can be predicted (11). Furthermore, near-infrared spectroscopy, particularly the thereby measured saturation of oxygen uptake in the skeletal muscle tissue, gives insight into the local perfusion and metabolic status (19–21). To date, such additional non-invasive and continuous approaches are still less applied during cardiopulmonary exercise testing. However, first results using these approaches to investigate central and peripheral factors of oxygen uptake simultaneously are promising (11).

It is clear, that age (22, 23), sex (23, 24), endurance capacity (23, 25), and chronic heart failure (11, 26) have an impact on maximum oxygen uptake and consequently also to the underlying central and peripheral factors. Previous studies investigating different age groups showed that children (22, 27) and older adults (23, 28) had lower values in central factors compared to young adults. Moreover, contradictory results were found concerning differences between younger and older adults on peripheral factors (21, 28). Regarding the differences between sexes, studies showed that male adults had higher values in central and peripheral factors than female adults (23, 24). Prior studies on endurance capacity revealed that trained adults possessed higher values in central (23, 25) but similar values in peripheral factors (23, 25, 28) compared to sedentary adults. Concerning chronic heart failure, it was shown that corresponding patients had lower values in central but similar values in peripheral factors compared to healthy controls (26, 29). However, most of these previous studies used invasive or non-continuous technologies. Such approaches are limited by the fact that they are not suitable for routine measurements due to the possible risk of infections or other complications (30, 31) as well as loss of information on physiological functions (32). Although previous studies built pioneer work in this research area, further studies with standardised, non-invasive, and continuous measurement approaches are needed to better understand differences in maximum oxygen uptake according to the underlying factors.

This study aimed to investigate the effects of age, sex, endurance capacity, and chronic heart failure on non-invasively and continuously measured central and peripheral factors of oxygen uptake. It was expected that there would be differences between the investigated groups assigned to effects. Our findings will increase the understanding of underlying factors of maximum oxygen uptake and may help to optimise specific diagnostics and interventions in sport science and medicine. Additionally, our study may indirectly question the validity of the non-invasive and continuous technologies and serve as an experimental framework for future studies. It should be noted that the data concerning chronic heart failure has already been published elsewhere (11) and was re-analysed here to associate with further reasonable populations.

## 2. Materials and methods

### 2.1. Participants

In total, 15 male children (11 ± 1 years), 15 male recreationally active adults (24 ± 3 years), 14 female recreationally active adults (23 ± 2 years), 12 male highly trained endurance athletes (24 ± 3 years), 10 male elders (59 ± 6 years), and 10 male chronic heart failure patients (62 ± 7 years) participated. For the examination of the four different effects, the groups were assigned as follows: (i) age: male children, male recreationally active adults, and male elders; (ii) sex: male and female recreationally active adults; (iii) endurance capacity: male recreationally active adults and male highly trained endurance athletes; and (iv) chronic heart failure: male elders (serving as healthy controls) and male chronic heart failure patients. The activity level of the recreationally active adults and the highly trained endurance athletes was defined according to the Participant Classification Framework (33). The participants were recruited from the sports students of the local university, different sports clubs, a local practice for cardiology, and the investigators' circle of acquaintances. Excluding the chronic heart failure patients, 25 different team or racquet (34%) and individual sports (66%) were performed by the participants, ranging from soccer, handball, and swimming to general fitness training, dancing, and horseback riding. Regarding the children, male, and female recreationally active adults, they were included in the study when they participated in 6 or less hours of sports per week, and their primary sport was not an endurance sport. The highly trained endurance athletes were included if they participated in cycling or triathlon competitions at least at a national amateur level. Concerning the elders and chronic heart failure patients, 3 or less hours of sports per week were allowed to participate. In case of the chronic heart failure patients, only 3 did any sport at all in form of cardiac rehabilitation (*n* = 1) and prevention programs (*n* = 2). Additionally, chronic heart failure patients were included when an ejection fraction of ≤35% was present to reduce the typical heterogeneity of chronic heart failure. Also, patients were individually medicated, with all taking beta-blockers. All participants stated that they had no acute or chronic diseases of the musculoskeletal system and those that militate against maximum exhaustion testing. Further descriptive characteristics on anthropometric characteristics and variables measured under maximum load of all six groups are presented in Table 1. All participants or, in the case of the children, the legal guardian signed a written informed consent. The study was approved by the Ethics Committee of the local university (MS/JE 29.11.11; MS/BB 180321).

**Table 1:** Anthropometric characteristics and variables measured under maximum load of the participants from six groups recruited.

Variables	Male children	Male recreationally active adults	Female recreationally active adults	Male highly trained endurance athletes	Male elders	Male chronic heart failure patients
	mean ± SD	mean ± SD	mean ± SD	mean ± SD	mean ± SD	mean ± SD
	(*n* = 15)	(*n* = 15)	(*n* = 14)	(*n* = 12)	(*n* = 10)	(*n* = 10)
Age [years]	11 ± 1	24 ± 3	23 ± 2	24 ± 3	59 ± 6	62 ± 7
Height [cm]	152 ± 6	186 ± 26	169 ± 6	183 ± 8	181 ± 9	178 ± 8
Body mass [kg]	42.2 ± 5.5	76.0 ± 6.2	62.2 ± 7.4	73.7 ± 8.1	90.7 ± 11.4	87.8 ± 11.7
BMI [kg/m^2^]	18.2 ± 2.0	23.6 ± 2.1	21.8 ± 1.8	21.9 ± 1.5	27.7 ± 2.6	27.7 ± 3.5
Fat mass [%]	20.4 ± 5.3	13.4 ± 3.3	22.4 ± 4.2	10.1 ± 2.4	25.1 ± 3.3	27.6 ± 3.4
Fat-free mass [kg]	33.4 ± 3.2	65.7 ± 3.9	48.1 ± 5.1	66.3 ± 7.7	67.8 ± 7.7	63.6 ± 7.2
Skinfold thickness^a^ [mm]	16 ± 6	8 ± 3	19 ± 5	4 ± 1	6 ± 2	9 ± 4
P_max_ [W]	140 ± 18	319 ± 25	210 ± 24	435 ± 39	201 ± 44	96 ± 33
VO_2rest_ [L/min]	0.4 ± 0.1	0.8 ± 0.3	0.5 ± 0.2	0.9 ± 0.3	0.6 ± 0.2	0.5 ± 0.2
VO_2max_ [L/min]	1.7 ± 0.2	3.7 ± 0.4	2.4 ± 0.3	4.4 ± 0.5	2.6 ± 0.5	1.4 ± 0.4
VO_2max_ [ml/min/kg]	41.1 ± 5.1	49.2 ± 5.8	39.4 ± 3.9	59.6 ± 3.5	28.6 ± 4.1	16.1 ± 5.9
HR_max_ [1/min]	187 ± 9	186 ± 7	184 ± 8	186 ± 6	156 ± 23	133 ± 16
RER_max_ [VCO_2_/VO_2_]	1.17 ± 0.05	1.26 ± 0.08	1.22 ± 0.08	1.30 ± 0.07	1.26 ± 0.07	1.12 ± 0.12
Lactate_max_ [mmol/L]	5.8 ± 1.5	11.6 ± 1.9	10.4 ± 1.7	11.9 ± 2.2	8.0 ± 3.1	4.4 ± 2.5
RPE_max_ [6–20]	20 ± 2	20 ± 0	20 ± 0	20 ± 1	20 ± 1	19 ± 1

SD, standard deviation; BMI, body mass index; P_max_, maximum power; VO_2rest_, oxygen uptake at rest; VO_2max_, maximum oxygen uptake; HR_max_, maximum heart rate; RER_max_, maximum respiratory exchange ratio; RPE_max_, maximum rating of perceived exertion.

^a^ Measured at right vastus lateralis muscle.

### 2.2. Study design

A cross-sectional design under laboratory conditions was applied. The measurements were carried out on a single day. The participants were instructed in advance not to eat any food and to only drink water for 2 h before testing. Furthermore, no strenuous exercises were allowed 24 h before the measurement. After anthropometric measures were taken, a cardiopulmonary exercise test until exhaustion on a cycling ergometer was performed. Here, the six aforementioned groups were the independent, while the dependent variables were as follows: oxygen uptake, systolic and diastolic blood pressure, mean arterial pressure, heart rate, stroke volume, cardiac output, cardiac power output, muscle oxygen saturation, and (calculated) arterio-venous oxygen difference. Importantly, in our study, the latter two dependent variables represented peripheral and the others central factors, as conducted before (11). During the measurements of the children and chronic heart failure patients a legal guardian and a cardiologist was present, respectively.

### 2.3. Anthropometric measurements

Using a 4-point bioelectric impedance analysis (Bodystat, Quadscan 4,000, Douglas, United Kingdom), body fat and fat-free mass were determined in supine position. Additionally, skinfold thickness of the right vastus lateralis muscle was measured using a calliper (Baseline® Medical Skinfold Caliper, Baseline® evaluation instruments, United States). The reason for determining the skinfold thickness was that interference with the near-infrared spectroscopy signal can occur due to the skin and subcutaneous tissue (34). The validity of the 4-point bioelectric impedance analyses is *r* = 0.98–0.99 (35).

### 2.4. Cardiopulmonary exercise test

The cardiopulmonary exercise test was performed on two different cycling ergometers. Generally, a stationary ergometer (Excalibur sport, Lode, Groningen, Netherlands) was used. To allow a more sport-specific testing for the highly trained endurance athletes, they brought their own bikes to be clamped in a mobile ergometer (Cyclus-2, RBM elektronik-automation, Leipzig, Germany). A ramp-like protocol was applied. For reaching comparable times to exhaustion, the initial as well as increasing load (every 60 s) was different between groups and conducted as follows: 5 W for chronic heart failure patients, 10 W for children and elders, 15 W for female recreationally active adults, 20 W for male recreationally active adults, and 30 W for highly trained endurance athletes. The load was aborted when the required pedalling frequency of 75–80 rpm could no longer be maintained (11). According to the initial loads of the corresponding test protocols, participants performed a 5-minute cool-down at 15, 30, 45, 60, and 90 W, respectively. To clarify exhaustion, three out of the following five criteria had to be reached, with only one being a subjective criterion: (i) heart rate ≥ 200 bpm-age; (ii) blood lactate ≥ 8 mmol/L; (iii) respiratory exchange ratio ≥1.1; (iv) rating of perceived exertion ≥19; and (v) visual analogue scale ≥60% of total exertion (36–38). However, there were adaptations for the children and chronic heart failure patients. Children had to reach two out of the following three criteria: (i) heart rate ≥95% of 200 bpm-age; (ii) blood lactate ≥ 4 mmol/L; and (iii) respiratory exchange ratio ≥1.0 (39, 40). Subjective criteria were not considered, as children tend to have a limited capacity for valid subjective assessment (41). For the chronic heart failure patients, clarification of exhaustion was solely based on subjective criteria, as conducted previously (11). During the cardiopulmonary exercise test, oxygen uptake was measured breath-by-breath using a gas analyser (Power-Cube Ergo, Ganshorn, Niederlauer, Germany). Prior to each testing, the system was calibrated according to the manufacturer's instructions. Additionally, every 60 s, systolic and diastolic blood pressure were measured manually on the left arm using a cuff and stethoscope (Boso Med-1, Bosch & Sohn, Jungingen, Germany). Equivalent mean arterial pressure was then calculated as described elsewhere (11). Immediately after reaching exhaustion, capillary blood was taken from the participant's earlobe to be analysed by an electro-enzymatic analyser (EKF-diagnostics, Biosen C_line Sport, Cardiff, United Kingdom) for the lactate concentration. The reliability of the stationary and mobile cycling ergometer, and gas and lactate analyser is CV = 8.2% (42), CV = 2.3% (43), ICC = 0.991–0.995 (44), and CV = 1.3% (45), respectively.

### 2.5. Bioreactance analysis

During the cardiopulmonary exercise test, non-invasively and continuously measured stroke volume and heart rate were recorded by a bioreactance analysis (Cheetah Nicom, Cheetah Medical, Vancouver, USA). The technology used is based on phase shifts, occurring when an alternating electric current with a frequency of 75 Hz is passed through the thorax. Then, stroke volume can be extrapolated from these phase shifts. Further details on the measurement principle can be found elsewhere (46). For measuring, eight electrodes were applied to the participant's back according to the manufacturer's instructions. Afterwards, cardiac output and cardiac power output, as the product of cardiac output and mean arterial pressure, were estimated as described elsewhere (11). All data were measured beat-by-beat. For the bioreactance analyses, validity and reliability are *r* = 0.82 (47) and ICC = 0.59–0.98 (48), respectively.

### 2.6. Near-infrared spectroscopy

Throughout the cardiopulmonary exercise test, muscle oxygen saturation of the right vastus lateralis muscle, as part of the main contribution to force production while cycling, was measured non-invasively and continuously by a near-infrared spectroscopy (Moxy Monitor, Fortiori Design LLC, Hutchinson, USA), as conducted previously (49). The technology is based on light waves ranging from 630 to 850 nm, which are sequentially being send from four light emitting diodes into the underlying tissue. The amount of returned scatted light is then recorded by two detectors. Further details on the measurement principle can be found elsewhere (49). According to the manufacturer's instructions, the near-infrared spectroscopy was surrounded by a light shield and then placed on the prominent part of the muscle belly of the vastus lateralis muscle. Data were measured at a frequency of 2 Hz. The reliability of the near-infrared spectroscopy is ICC = 0.77–0.99 (49). Additionally, as a peripheral factor, the arterio-venous oxygen difference was calculated from oxygen uptake and cardiac output using the Fick's principle.

### 2.7. Statistical analysis

For statistical analyses, all data collected during the cardiopulmonary exercise test were averaged over 60 s to fit its incremental stages. Then, they were considered at rest and interpolated at 80% and 100% of maximum oxygen uptake. For descriptive purposes, the relative oxygen uptake at all interpolated points was presented as well. The normal distributions of the data were checked by the Shapiro-Wilk test. A two-factor repeated measure ANOVA (group × time) was applied to examine the effects of age, sex, endurance training, and chronic heart failure. The assumption of sphericity was checked with the Mauchly's test and, if required, the degrees of freedom were adjusted using the Greenhouse–Geisser correction. Effect sizes were calculated using partial eta-squared ($\eta_{p}^{2}$), with ≥.01 indicating small, ≥.059 medium, and ≥.138 large effects (50). Differences at rest, 80, and 100% of maximum oxygen uptake were calculated by post-hoc tests using Bonferroni correction. To increase the readability, the data are only presented in figures. The precise values can be calculated from the raw data provided in the Supplementary Material. For the discussion on central and peripheral factors of oxygen uptake, only the data provided by the figures and tested by the aforementioned statistical analyses were used.

## 3. Results

### 3.1. Age

**Figure 1:**
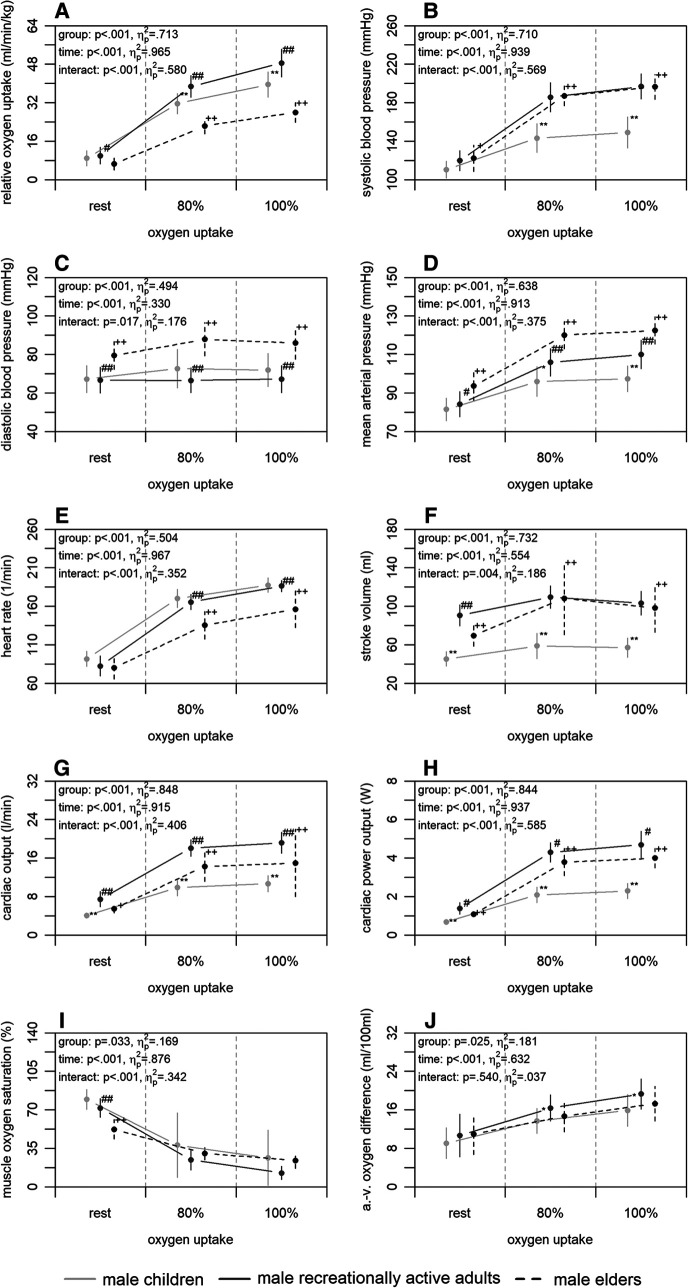
Relative oxygen uptake (A), central (B–H), and peripheral factors (I, J) of the male children, recreationally active adults, and elders at rest, 80, and 100% of maximum oxygen uptake. a.-v., arterio-venous. Means and standard deviations are shown. The asterisks * and ** indicate differences between children and adults at *p* < .05 and <.001, respectively. The hashes # and ## indicate differences between adults and elders at *p* < .05 and <.001, respectively. The plus signs + and ++ indicate differences between elders and children at *p* < .05 and <.001, respectively.

Figure 1 shows the mean differences in central and peripheral factors of oxygen uptake between male children, recreationally active adults, and elders. There were statistically significant group differences (*p* ≤ .033) supported by large effect sizes ($\eta_{p}^{2}\geq .169$) for all factors. Time differences were also statistically significant (*p* < .001) with large effect sizes ($\eta_{p}^{2}\geq .330$) for all factors. Interaction differences were statistically significant for all factors (*p* ≤ .017) supported by large effect sizes ($\eta_{p}^{2}\geq .176$), with exception of the arterio-venous oxygen difference (*p* = .540; $\eta_{p}^{2}=.037$).

### 3.2. Sex

**Figure 2:**
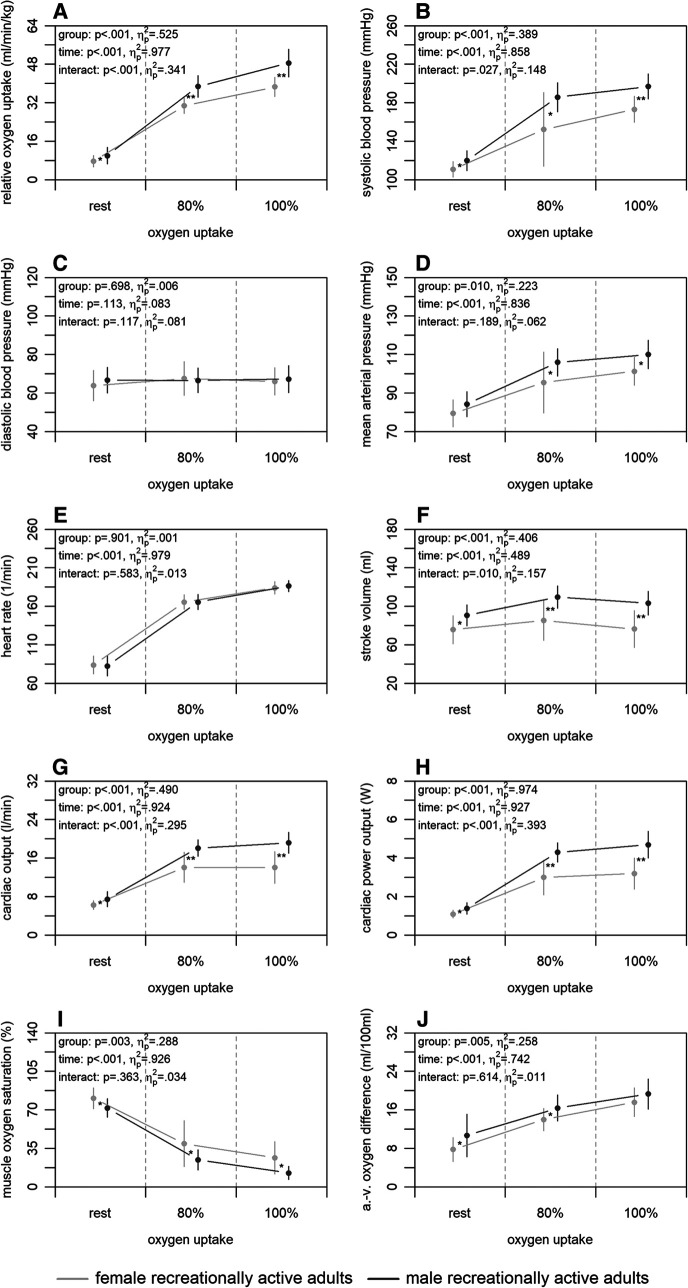
Relative oxygen uptake (A), central (B–H), and peripheral factors (I, J) of the male and female recreationally active adults at rest, 80, and 100% of maximum oxygen uptake. a.-v., arterio-venous. Means and standard deviations are shown. The asterisks * and ** indicate differences at *p* < .05 and <.001, respectively.

Figure 2 shows the mean differences in central and peripheral factors of oxygen uptake between male and female recreationally active adults. There were statistically significant group differences for all factors (*p* ≤ .010) with large effect sizes ($\eta_{p}^{2}\geq .223$), except for the diastolic blood pressure and heart rate (*p* ≥ .698; $\eta_{p}^{2}\leq .006$). Time differences were statistically significant for all factors (*p* < .001) supported by large effect sizes ($\eta_{p}^{2}\geq .489$), with exception of the diastolic blood pressure (*p* = .133; $\eta_{p}^{2}=.083$). Interaction differences were statistically significant for the systolic blood pressure, stroke volume, cardiac output, and cardiac power output (*p* ≤ .027) supported by large effect sizes ($\eta_{p}^{2}\geq .148$).

### 3.3. Endurance capacity

**Figure 3:**
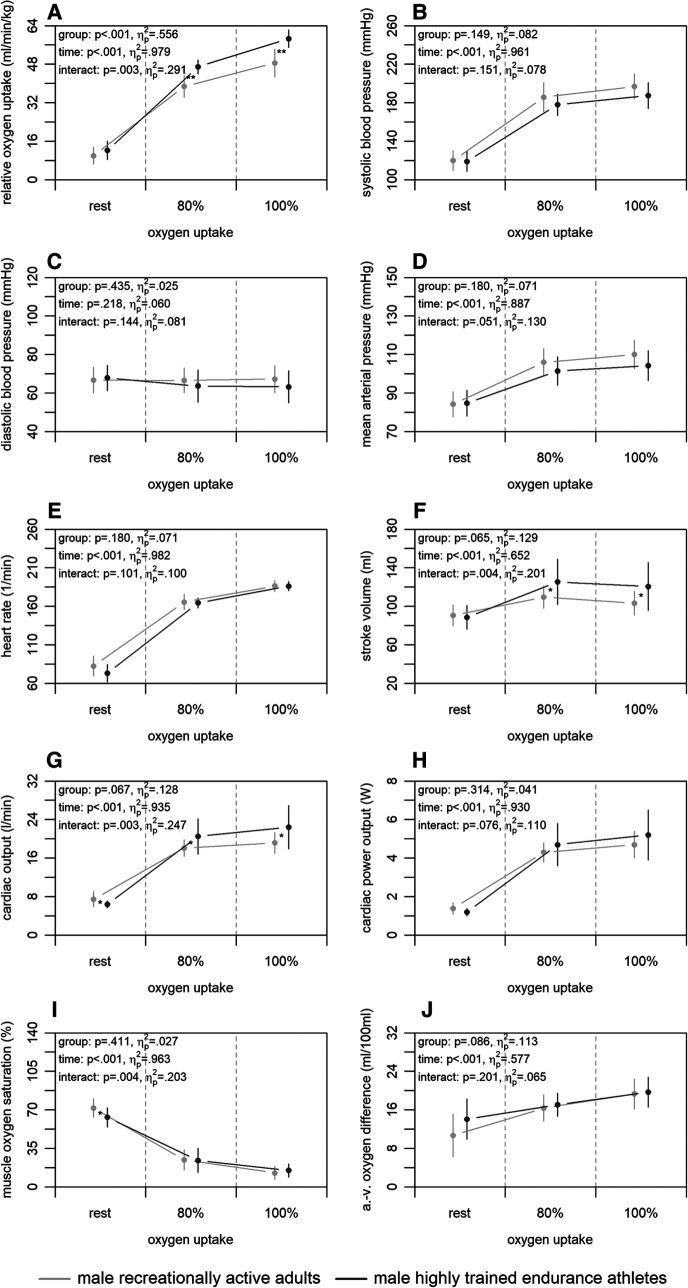
Relative oxygen uptake (A), central (B–H), and peripheral factors (I, J) of the male highly trained endurance athletes and recreationally active adults at rest, 80, and 100% of maximum oxygen uptake. a.-v., arterio-venous. Means and standard deviations are shown. The asterisks * and ** indicate differences at *p* < .05 and <.001, respectively.

Figure 3 shows the mean differences in central and peripheral factors of oxygen uptake between highly trained endurance athletes and male recreationally active adults. There were no significant group differences for any of the factors (*p* ≥ .065) albeit up to medium effect sizes ($\eta_{p}^{2}\leq .129$). Time differences were statistically significant for all factors (*p* < .001) with large effect sizes ($\eta_{p}^{2}\geq .577$), with exception of the diastolic blood pressure (*p* = .218; $\eta_{p}^{2}=.060$). Interaction differences were statistically significant for the stroke volume, cardiac output, and muscle oxygen saturation (*p* ≤ .004) supported by large effect sizes ($\eta_{p}^{2}\geq .201$).

### 3.4. Chronic heart failure

**Figure 4:**
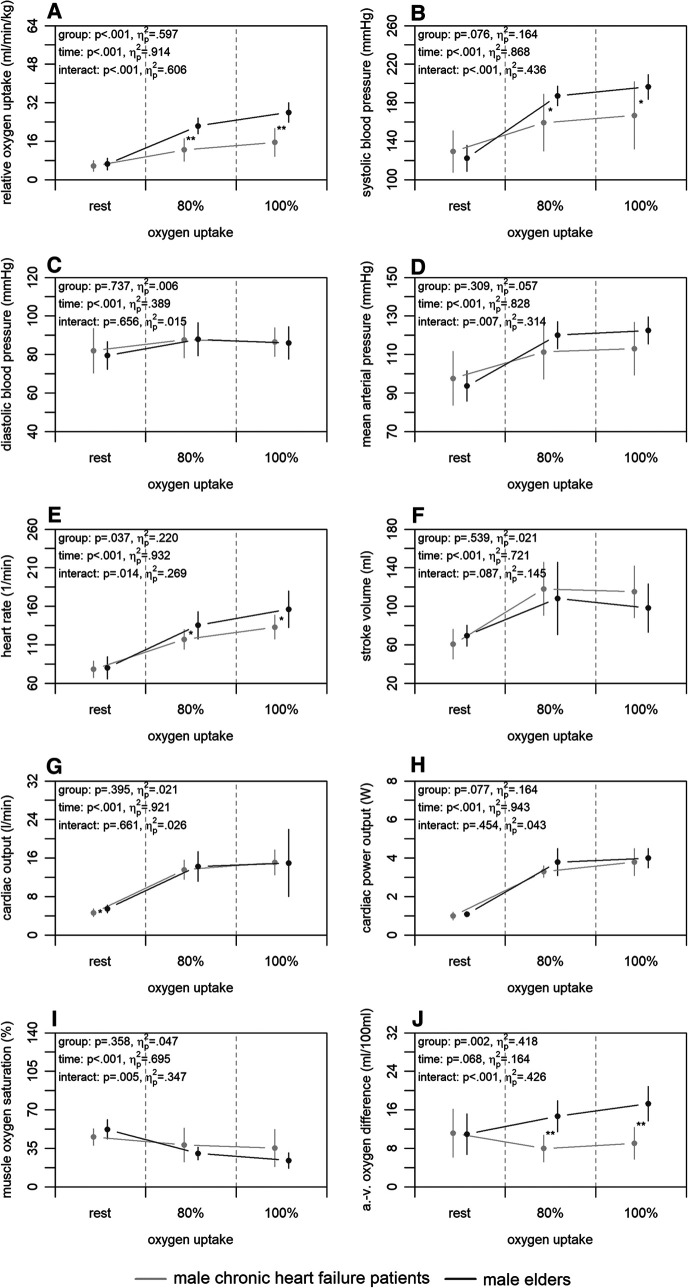
Relative oxygen uptake (A), central (B–H), and peripheral factors (I, J) of the male chronic heart failure patients and elders at rest, 80, and 100% of maximum oxygen uptake. a.-v., arterio-venous. Means and standard deviations are shown. The asterisks * and ** indicate differences at *p* < .05 and <.001, respectively.

Figure 4 shows the mean differences in central and peripheral factors of oxygen uptake between male chronic heart failure patients and elders. There were statistically significant group differences for heart rate and arterio-venous oxygen difference (*p* ≤ .037) with large effect sizes ($\eta_{p}^{2}\geq .220$). For all factors, time differences were statistically significant (*p* < .001) supported by large effect sizes ($\eta_{p}^{2}\geq .389$), with exception of the arterio-venous oxygen difference (*p* = .068; $\eta_{p}^{2}=.164$). Interaction differences were statistically significant for the systolic blood pressure, mean arterial pressure, heart rate, muscle oxygen saturation, and arterio-venous oxygen difference (*p* ≤ .014) supported by large effect sizes ($\eta_{p}^{2}\geq .269$).

## 4. Discussion

As far as we know, this is the first study to give an experimental framework of the effects of age, sex, endurance capacity, and chronic heart failure on non-invasively and continuously measured central and peripheral factors of oxygen uptake by the same standardised methodological approach. Our study revealed new insights into the relationship and differences between maximum oxygen uptake and underlying factors in six different groups and may help to optimise specific diagnostics and interventions in sport science and medicine as well as indirectly question the validity of the used non-invasive and continuous technologies. The main findings were that (i) between children, adults, and elders, central factors of oxygen uptake differed, whereas peripheral factors were similar with exception of the comparison between children and adults; and (ii) male and female adults showed differences in both central and peripheral factors. Also, (iii) male highly trained endurance athletes indicated higher central factors compared to male recreationally active adults, while peripheral factors were similar; and (iv) patients with chronic heart failure had similar values in central factors compared to elders, but peripheral factors differed.

### 4.1. Age

Concerning our first main finding, non-invasively and continuously measured central factors of oxygen uptake differed between age groups, while peripheral factors were similar with exception of the comparison between children and adults. Compared to the children, the recreationally active adults showed a higher relative maximum oxygen uptake (Figure 1A). This finding was accompanied by a higher stroke volume, cardiac output, and cardiac power output of the adults at 100% of maximum oxygen uptake (Figure 1F–H), as reported previously using a non-continuous rebreathing technique or transthoracic echocardiography (22, 27). Therefore, the higher maximum oxygen uptake of the adults could be due to the higher cardiac output and this in turn to the higher stroke volume compared to the children. These findings are plausible, as they are possibly caused by the generally larger heart volume of adults (51, 52). Furthermore, the adults showed a higher maximum arterio-venous oxygen difference and a tendency of a lower muscle oxygen saturation at 100% of maximum oxygen uptake (Figure 1I,J). These outcomes are also in line with previous studies calculating peripheral resistance (22, 27). The differences may possibly be caused by a higher haemoglobin concentration of the adults (51, 52) on the one hand. On the other, they could be due to differences in the ability of myoglobin to store oxygen or to facilitate oxygen diffusion into the mitochondria (53). Regardless of the reason, which cannot be precisely identified here, the findings indicate that the peripheral utilisation of oxygen seems to be limited in prepubescent age, requiring more research to clarify. The second comparison regarding the effect of age was between adults and elders. The results showed a higher relative maximum oxygen uptake of the adults (Figure 1A). Complementary, the adults had a higher heart rate, cardiac output, and cardiac power output at 100% of maximum oxygen uptake (Figure 1E,G,H). Previous studies also reported these findings applying non-continuous rebreathing techniques (23, 54); however, one additionally stated a lower stroke volume in younger compared to older adults (23). It is known that with increasing age, maximum heart rate decreases, which is an inevitable and irreversible process (55). Therefore, it can be concluded that the lower maximum heart rate of the elders resulting in a lower cardiac output is the main reason for the lower maximum oxygen uptake compared to adults. This can be assumed, since the peripheral factors of both groups were similar at 100% of maximum oxygen uptake (Figure 1I,J). Former studies calculating peripheral factors or using the invasive cyanmethemoglobin method also revealed these findings (23, 28). However, one study using near-infrared spectroscopy showed a higher peripheral utilisation of oxygen in younger compared to older adults (21). This could be due to a loss of capillaries and a decrease in volume density of the mitochondria and their enzyme activity in the muscle with increasing age (51, 52). The explanations given here for the age effect, are again confirmed by the last comparison between children and elders. The elders had a lower relative maximum oxygen uptake (Figure 1A). However, the elders showed a higher stroke volume, cardiac output, and cardiac power output but lower heart rate as well as similar peripheral factors at 100% of oxygen uptake (Figure 1E–J). Taken together, mainly central factors, and in adulthood especially heart rate, are influenced by age, whereas there only seems to be a small effect on the peripheral factors of oxygen uptake.

### 4.2. Sex

Our second main finding was that male and female recreationally active adults differ regarding non-invasively and continuously measured central and peripheral factors of oxygen uptake. The comparison showed a higher absolute and relative maximum oxygen uptake of the male adults (Figure 2A). At central level, males had a higher stroke volume, cardiac output, and cardiac power output at 100% of maximum oxygen uptake (Figure 2F–H). Similar results were also shown by a previous study using a non-continuous rebreathing technique (23), which detected a higher stroke volume and cardiac output in men compared to women, independent of age. It is morphologically known that males on average have a disproportionally larger heart compared to females (56), which may be the cause of the detected differences in stroke volume and cardiac output. The possible higher efficiency of the heart in males is supported by their equally higher cardiac power output (Figure 2H), even though females tend to have a higher ejection fraction (56). Additionally, a study (23) relativised stroke volume to fat-free mass to compensate for group differences. Doing the same with the results of the present study, it shows that the fat-free mass might be decisive for the higher stroke volume of the males (data not shown). Regarding the peripheral factors, our findings showed lower muscle oxygen saturation and higher arterio-venous oxygen difference of the males at 100% of maximum oxygen uptake (Figure 2I,J). Current literature calculating peripheral factors shows contradictory results (23, 24). However, these studies were conducted at submaximal load only or on a treadmill and may therefore not be completely comparable to our study. Nevertheless, our results suggest a higher peripheral utilisation of oxygen in males, which may result from the higher haemoglobin concentration (51, 52) or differences in myoglobin functions (53) compared to females.

### 4.3. Endurance capacity

The third main finding was that male highly trained endurance athletes had higher non-invasively and continuously measured central factors of oxygen uptake compared to recreationally active adults, while peripheral factors were similar. Here, the athletes showed a higher relative maximum oxygen uptake (Figure 3A). This was accompanied by a higher stroke volume and cardiac output, while heart rate and cardiac power output were similar at 100% of maximum oxygen uptake (Figure 3E–H). Earlier studies using non-continuous rebreathing techniques support these findings (23, 25). Only one study also applying a non-continuous rebreathing technique (28) did not detect differences in central factors between competitive and recreational runners. However, the study was conducted in a submaximal manner and the authors extrapolated the findings to a maximal setting. The results presented in our study may be caused by a physiologic hypertension of the heart muscle by endurance training (57). Furthermore, both groups showed similar muscle oxygen saturation and arterio-venous oxygen difference at 100% of maximum oxygen uptake (Figure 3I,J), which is also in line with former studies calculating peripheral factors or using the invasive cyanmethemoglobin method (23, 25, 28). Therefore, peripheral factors do not seem to be responsible for the higher oxygen uptake of the highly trained endurance athletes, even though previous studies show that endurance training leads to a capillarisation and an increase in volume density of mitochondria and their enzyme activity in the muscles (58, 59). Nevertheless, endurance training seems to have an effect on peripheral factors in females and elders (23), which was, however, not investigated in the current study.

### 4.4. Chronic heart failure

Concerning our last main finding, the chronic heart failure patients had similar values in non-invasively and continuously measured central factors of oxygen uptake compared to elders, but peripheral factors differed. Between both groups, the chronic heart failure patients showed a lower relative maximum oxygen uptake in patients (Figure 4A). At central level, only differences in heart rate, which was lower in patients, were detected at 100% of maximum oxygen uptake (Figure 4E). This is plausible as the lower heart rate may be caused by the intake of betablockers (60). Generally, our results on central factors differ from those revealed in previous studies. Two studies using a non-continuous rebreathing technique or transthoracic echocardiography (26, 29) showed lower stroke volume and cardiac output in chronic heart failure patients under maximum load. Yet, the ejection fraction of patients in both studies was similar to that in our patients and thus does not seem to be the reason for the inconsistent results. Regarding the peripheral factors, chronic heart failure patients showed lower arterio-venous oxygen difference and a tendency of muscle oxygen saturation being higher compared to elders at 100% of maximum oxygen uptake (Figure 4I,J). Again, these results are not in line with those of a former study applying catheters (26). Our results show a possibly lower peripheral oxygen utilisation of the chronic heart failure patients, which may be caused by effects such as muscle atrophy, reduction of mitochondrial density, and decrease of mitochondrial enzyme activity (61, 62) as well as reduced capillary density (63). These overall contradictory results throughout the literature illustrate that chronic heart failure cannot be generalised and depends on many other intra- and interindividual factors (64). Both central and peripheral factors can have an influence on the reduced maximum oxygen uptake in chronic heart failure patients regardless of their ejection fraction. Such knowledge can be used for exercise prescription (65), for which more interventional research is required.

## 5. Limitations

While our study increases the knowledge on central and peripheral factors of oxygen uptake, few limitations should be acknowledged. Firstly, the reliability of the applied bioreactance analysis and near-infrared spectroscopy decreases with increasing load (48, 49), which may have had an impact on our outcomes. Especially in the context of the applied bioreactance analysis, as a non-invasive and continuous approach to measure cardiac output, its validity is still controversially discussed (66). Although the implemented bioreactance analysis shows acceptable validity compared to thermodilution catheter in patients (47), more evaluation studies across different exercise modalities and loads are needed. Secondly, our study is limited by a not *a priori* conduced statistical power analysis and small sub-group sample sizes (*n* = 10–15) being mostly underpowered. However, in total, 76 participants were investigated by a standardised measurement approach. Additionally, effect sizes being independent of sample size were calculated. More research evaluating both non-invasive technologies in larger groups is needed. Thirdly, concerning the age and sex effect, we did not consider the biological age and menstrual cycle, respectively. This may have had an impact on our results and should be looked at in future studies for which our study can serve as a framework. Lastly, there is an ongoing discussion regarding criteria to clarify exhaustion (67). In our study, we carefully applied and modified the criteria of exhaustion for the different groups of participants from previous research (36–40). Based on the raw data during maximum load (Supplementary Material), we are sure that only exhausted participants were considered.

## 6. Conclusion

The present study shows that age, sex, endurance capacity, and chronic heart failure affect central and peripheral factors of oxygen uptake measured by non-invasive and continuous technologies. Since most of our findings support pioneer work using invasive or non-continuous measures, the validity of our applied technologies is indirectly confirmed. Our outcomes allow direct comparison between different groups serving as reference data and framework for subsequent studies in sport science and medicine aiming to optimise diagnostics and interventions in athletes and patients.

## Data Availability

The original contributions presented in the study are included in the article/**Supplementary Material**, further inquiries can be directed to the corresponding author.
